# Development of an autonomous flow-proportional water sampler for the estimation of pollutant loads in urban runoff

**DOI:** 10.1007/s10661-020-08536-3

**Published:** 2020-08-08

**Authors:** Péter Budai, Máté Krisztián Kardos, Marcell Knolmár, Gábor Szemán, József Turczel, Adrienne Clement

**Affiliations:** 1 Budapest University of Technology and Engineering, Műegyetem rkp. 3, Budapest, 1111 Hungary; 2 Miskolc, Hungary; 3 Budapest, Hungary

**Keywords:** Urban runoff, Flow-proportional, Event-based, Water sampling, Micropollutants

## Abstract

Implementation of an extensive urban runoff monitoring program, targeting the quantification of heavy metal and organic micropollutant loads, necessitated the development of an autonomous water sampler. The design requirements for the device were to fulfill flow-proportional continuous composite sampling of urban runoff events in a widely customizable, relatively inexpensive, and simple way. In this paper, we introduce the concept along with the experiences gained from the first several months of field tests at seven pilot areas in Hungary that represent a wide range of urban environments. During the test period, prototype samplers were placed in natural (urban) streams as well as stormwater drainage pipes, resulting in a total of 97 automatic composite runoff samples. At two sites, an additional 28 manual grab samples were collected to represent time series from five distinct runoff events. Sampling efficiency was checked by comparing collected volumes with the theoretical ones (derived from pump mileage data). Ranges and ratios of concentrations measured from composite and grab samples were graphically interpreted in order to evaluate their representativeness. It has been shown that the concept is suitable for conducting cost-effective urban runoff characterization surveys targeting inter-event variability.

## Introduction

As the share of population living in cities is steadily increasing globally, issues regarding the urban water cycle are gaining attention worldwide. An important aspect of this topic is the deteriorating quality of surface waters receiving pollution from the built-up lands. Contamination of urban stormwater originates from various sources such as atmospheric deposition, dissolution from roof and facade materials, as well as emissions from transportation and wastewater misconnections (Müller et al. 2020). While point source pollution (e.g., wastewater network discharges or combined sewer overflows) is relatively easy to localize and thus to quantify and mitigate, this is not true for water-polluting anthropogenic activities that spread over large and diverse urban areas (Kardos and Clement 2020). Pollution originating from diffuse sources is of higher variability because it is related to runoff generated by occasional and stochastic precipitation events of varying scale (Budai and Clement 2007; Kovács and Clement 2009; Stutter et al. 2017). The quantification of such pollutant loads in urban water bodies is thus often based on event-based sampling campaigns; however, very different methodological approaches exist in practice.

The easiest and most traditional sample acquisition method is simple manual grab sampling, when personnel travels to the sampling location at a given point of time, fills the desired number and type of previously prepared bottles with the sampled media, and takes them to the lab where the samples get analyzed for the constituents in question. For regular monitoring purposes, this method is often sufficient. For compounds that are continuously present in a relatively permanent manner, it might even allow for the quantification of load values passing through the particular section with satisfactory reliability (Clement 2001; Lee et al. 2016; Stenback et al. 2011). However, this strategy fails to be informative in the case of rapidly changing constituent concentrations that coincide with short-term flood/runoff events (e.g., Lee et al. 2007). One kind of workaround, often applied in urban rainfall runoff studies (e.g., Chow et al. 2013; Fu et al. 2018; Lee et al. 2002; Li et al. 2015; Li et al. 2006; Li et al. 2012; Shinya et al. 2000; Wang et al. 2017), is to maintain presence over the entire event and take several samples according to a predefined plan. However, the conditions for this method are not always favorable (timely arrival to the site cannot be guaranteed, and round-the-clock availability of trained workforce is also limited).

Automatic sample acquisition methods are more sophisticated, as they eliminate the need for human interaction during the events. Such devices can be classified into two main groups, depending on whether any decision or action has to be taken to start or terminate the sampling process. Solutions that do not require any intervention are referred to as passive methods. Passive automatic samplers collect either water samples or only selected contaminants (e.g., via their special shape or material) simply owing to their placement. For roof or road runoff, bottles or containers can be mounted into an appropriate point of the runoff routing system (gutter, gully, shoot) and will thus be automatically filled with usually the most contaminated part: the so-called first flush of a rain event (Bäckström et al. 2003; Budai and Buzás 2007; Helmreich et al. 2010; Horváth 2011; Mangani et al. 2005; Robertson et al. 2019). In smaller creeks, grits or meshes can be placed into the river course; and by setting their level, they can also be linked to specific high-flow conditions (Flödl et al. 2020). In the case of larger streams, passive samplers are usually mounted on buoys, ships, or artificial buildup parts of the riverbed (Lindim et al. 2016; Miège et al. 2015; Vrana et al. 2014).

The most advanced approach uses active automatic devices, where the sampling process is triggered by some continuously measured characteristic of the sampled media, such as its water level or flow (e.g., Lessels and Bishop 2020; Walling and Teed 1971), electric conductivity (e.g., Gromaire-Mertz et al. 1998), or turbidity (e.g., Lewis 1996). When the value of the permanently measured parameter falls below (in the case of electric conductivity) or exceeds (in the case of the other parameters) a certain threshold (e.g., 95% percentile stream level), a pump is activated, conveying a portion of the sampled media into the sampling vessel(s), with optional filtering in between. The individual samples might be collected over short time intervals (resulting in discrete automatic grab samples) or over a longer time span (ending up with a composite sample). Commercially available instruments usually gather a series of discrete grab samples that are pumped at predefined, but not necessarily equal flow or time intervals (e.g., Gan et al. 2008; Han et al. 2006; Leecaster et al. 2002; Lee et al. 2011). In some cases, turbidity might also be taken into account (Lewis 1996). The series of samples can then either be analyzed one by one or—considering the continuous flow records—combined into one or more composite samples (Becouze-Lareure et al. 2019; Lessels and Bishop 2020; Liu et al. 2013). When taking composite samples, the continuously measured parameters (such as the flow) might be used to adjust the sampling rate (e.g., Kayhanian et al. 2007; LaBarre et al. 2016; Sabin et al. 2005). Sampling methods such as the collection of pollutants from impervious urban surfaces by sweeping or washing (e.g., Budai and Clement 2018; Vaze and Chiew 2002) and by vessels mounted on moving cars (Budai and Clement 2012) can be viewed as special, targeted cases of active sampling.

The choice of sampling strategy depends on the aim of the campaign (Huber et al. 2016; Kozak et al. 2019), along with the project budget. If the goal is to gain information on the intra-event dynamics, a series sampler is needed and many samples will have to be analyzed. However, for the quantification of inter-event variability, composite samplers are sufficient, on condition that they are capable of adjusting the pumping rate to be proportional with the flow during the sampling process. The concentration of polluting substances in such samples will represent the so-called event mean concentration (EMC) which is a widely accepted parameter used to characterize unique events (Göbel et al. 2007; Li et al. 2012; Opher and Friedler 2010). As flow-proportional composite samples inherently yield the EMCs (Gasperi et al. 2014; Sabin et al. 2005), they allow for the easy calculation of the total event loads as well (by simple multiplication with the total event runoff).

A selection of different automatic samplers are available on the market; however, their price or some of the technical limitations might pose difficulties for particular research aims. This was the case for an extensive monitoring program, targeting the quantification of heavy metal and organic micropollutant event loads in urban runoff flows at several different sites concurrently, where the following project aims were addressed:

- Continuous water level monitoring and recording in user-defined time intervals
- Sampling driven by a flexible, user-specified program, based on actual measured water levels
- Inexpensive and simple system parts that are easily replaceable or repairable on-site
- Low-energy consumption (in order to enable off-grid deployment)
- Weatherproof system design

In this paper, we introduce the concept and the first prototypes of a widely customizable autonomous flow-proportional water sampler that was designed to fulfill the requirements outlined above, along with the experiences gained from the first several months of field tests.

## Materials and methods

### Hardware layout

**Fig. 1:**
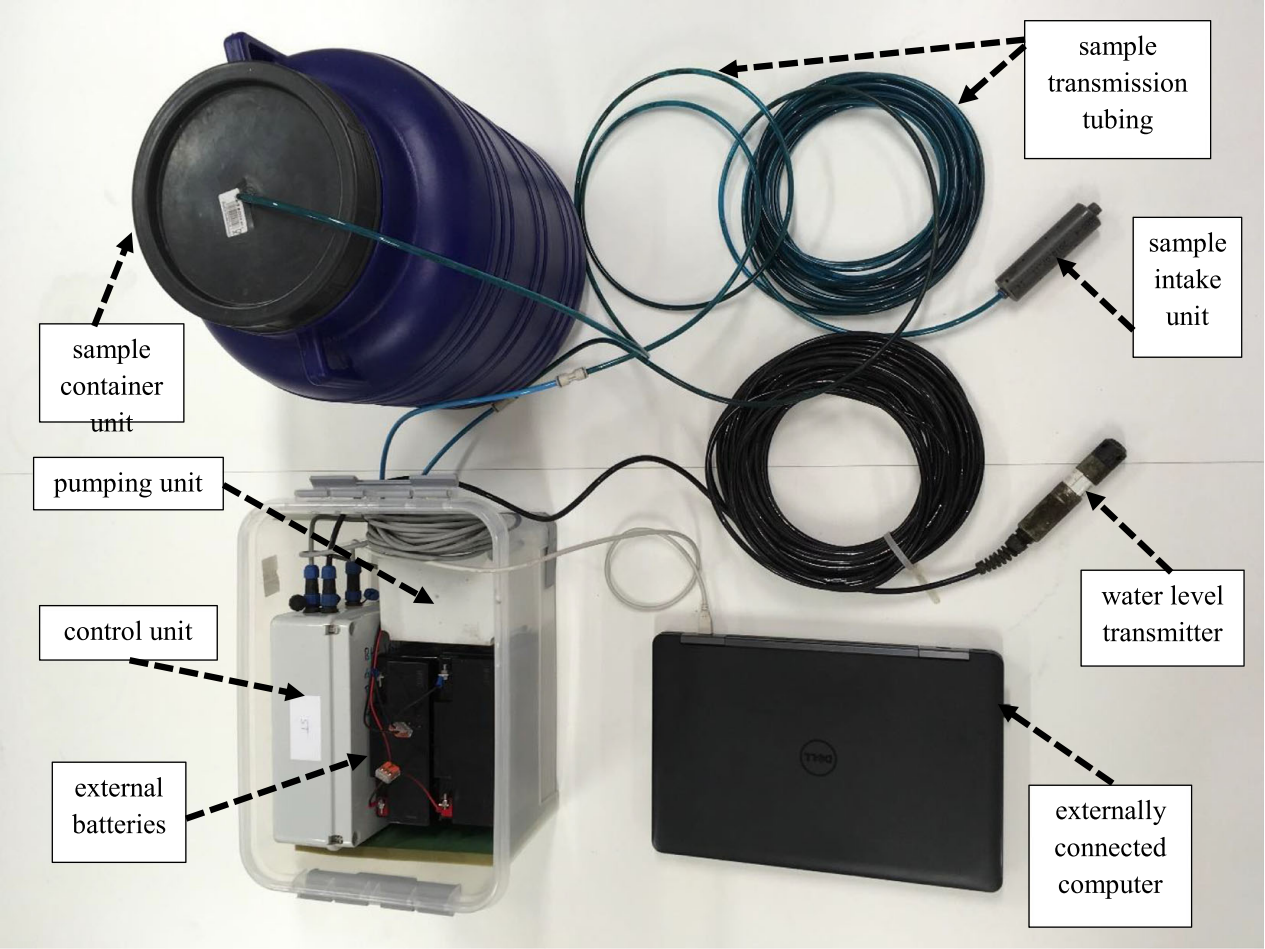
Building blocks of the modular sampling assembly

In order to keep the equipment flexible enough for deployment in various field situations, a modular approach was adopted, so that the arrangement of the system units can always be adapted to the layout of the actual site. The developed sampling assembly consists of the following elements (Fig. 1):

- Water level transmitter (WLT)
- Pumping unit (PU)
- Control unit (CU) with exchangeable internal battery
- Sample collection and transmission system parts (intake unit, tubes, and sample container)
- Communication cables between (i) the WLT and the CU, (ii) the PU and the CU, and (iii) the CU and any externally connected computer (for on-site setup, updates, and data download)
- Exchangeable external batteries

Water level was chosen as commanding parameter because of its simple measurement methodology and relatively carefree operation in the long term, compared with, e.g., flow rate or turbidity meters. In addition, the price range of reliable WLT products is more affordable than the other alternatives. In the prototypes, three different commercially available analogue WLT models were tested and used, whose technical details are presented in Table 1. Nevertheless, the system can work with other types of analogue WLT products as well.

**Table 1:** Specifications of the water level transmitters used in the prototypes

Product and model	Dataqua DA-LUB 450	SPK SPW03-M1D1Z	Qidian QDY30A
Output signal	0…5 V DC	4…20 mA	4…20 mA
Measuring range	0…4 m	0...5 m	0...5 m
Accuracy	± 0.4%	± 1.5%	± 0.5%
Power supply	12 V DC	12 V DC	12 V DC
Length of data cable	20 m	6 m	15 m
Probe protection	IP68	IP65	IP68

**Fig. 2:**
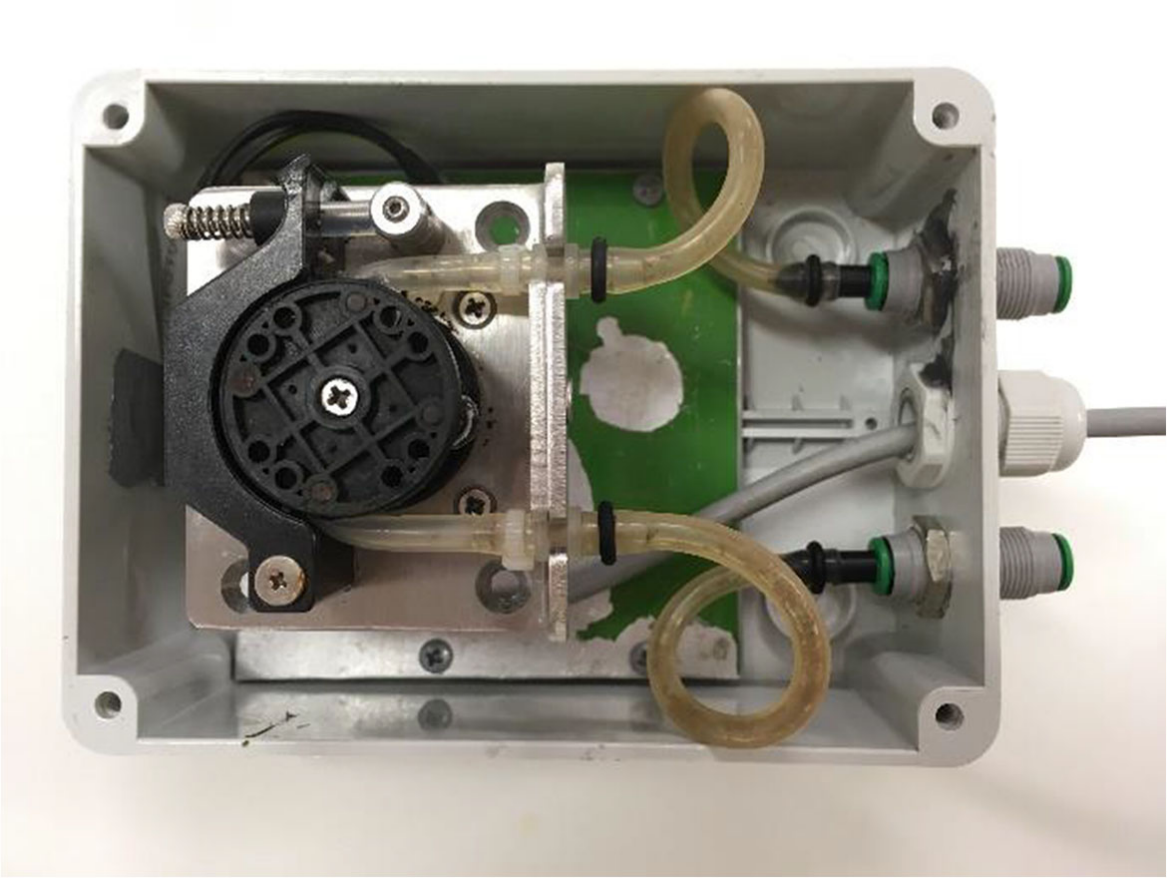
Internal view of the pumping unit box (lid removed)

The prototype PU consists of a Kamoer KCS PLUS-SM-4-B10 peristaltic water pump (driven by a 12 V DC stepper motor), built into an IP56 casing (Fig. 2). The internal tubing of the pump can be manually dismounted for cleaning and replacement, if necessary. The achievable maximal sampling rate (up to 170 mL/min) depends on mechanical settings within the pump (the position of the clamp that ties down the internal tube is adjustable) and the height difference between the pump and the sampled water level. Thus, the working range of the pump can be tailored individually for each application, upon installation at the actual site. During sampling, the flow rate is controlled by adjusting the rotation speed to the desired level, depending on the changes in the measured water level. This task is handled by the CU.

**Fig. 3:**
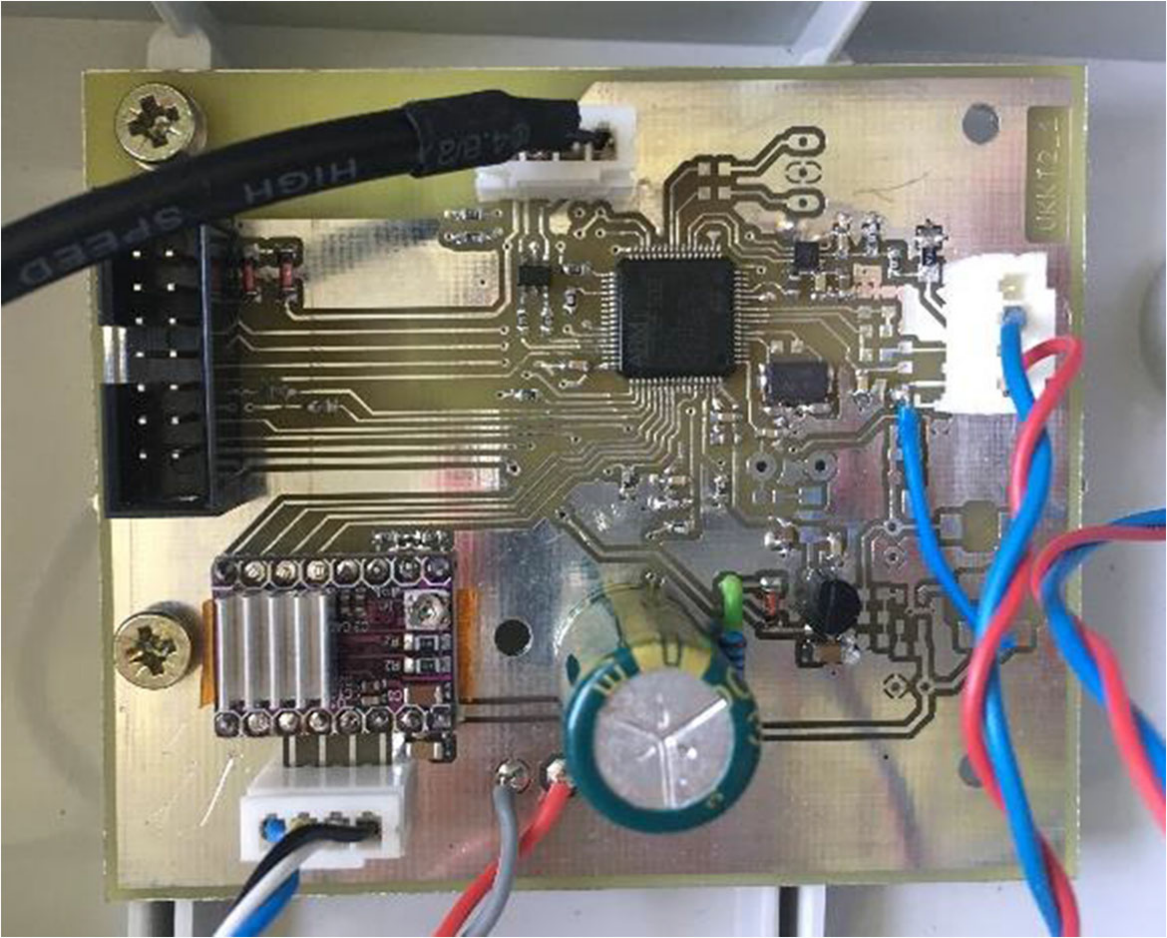
The printed circuit board that forms the principal component of the control unit

**Fig. 4:**
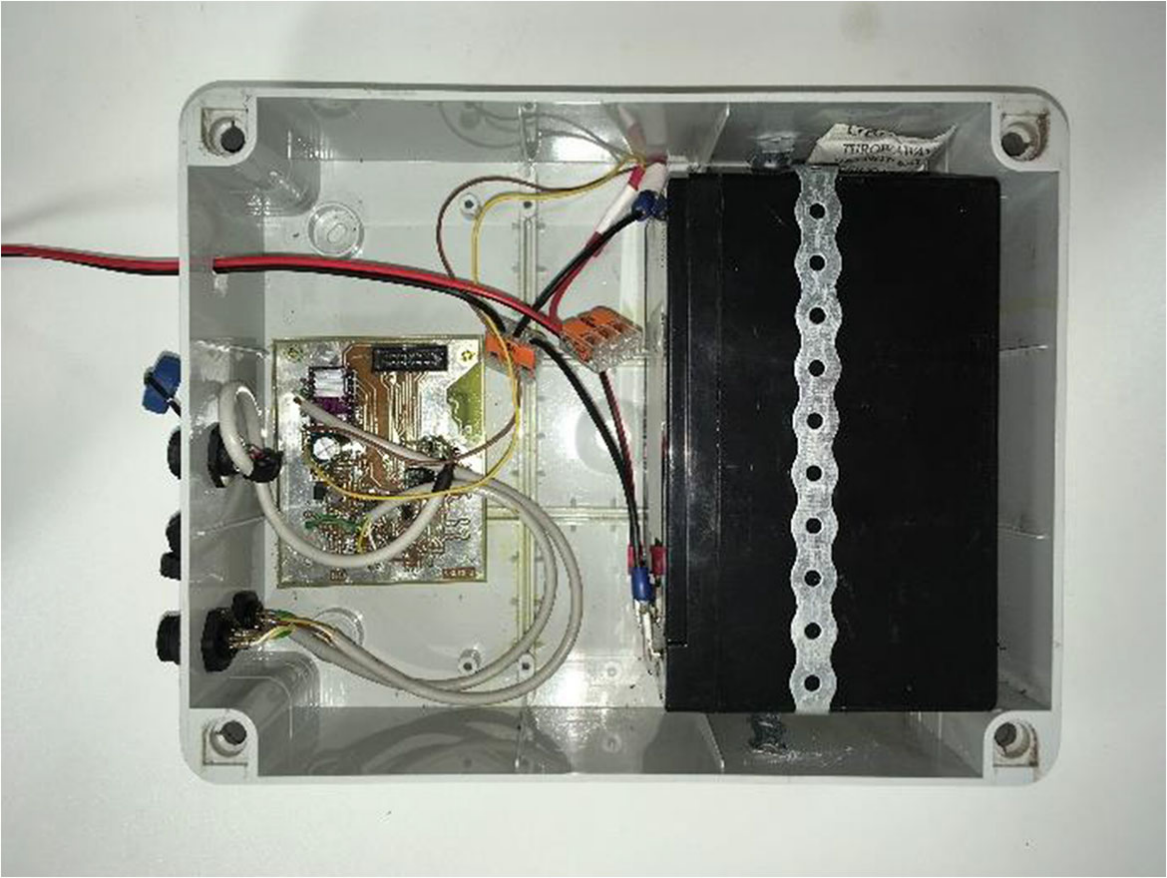
Internal view of the control unit box (lid removed)

The CU hosts a custom-designed printed circuit board that contains an STM32F103 microcontroller, an A4988 stepper motor driver, and an RF-BT0417C wireless TTL transceiver module. This board (shown on Fig. 3) takes care of (i) processing, registering, and storing the incoming signals from the WLT; (ii) sending out commanding signals to the PU via the stepper motor driver; (iii) communication with the user program, as well as (iv) managing the power supply of the WLT and PU. The board itself is protected against moisture condensation and is contained by an IP56 casing box (Fig. 4). Communication between the CU and an external computer can be established by a USB cable, as well as a custom-designed serial cable (used for executing firmware updates, if necessary) and via Bluetooth (for on-site wireless control).

Since autonomous operation is a key factor for most of the potential applications, the system was designed with low power consumption in mind. Power is provisioned by a rechargeable 12 V battery set consisting of one 9 Ah capacity unit as internal power source, stored within the CU (Fig. 4), and two additional 18 Ah capacity units as external power sources. The latter are more easily accessible and can be exchanged without dismantling the case of the CU.

**Fig. 5:**
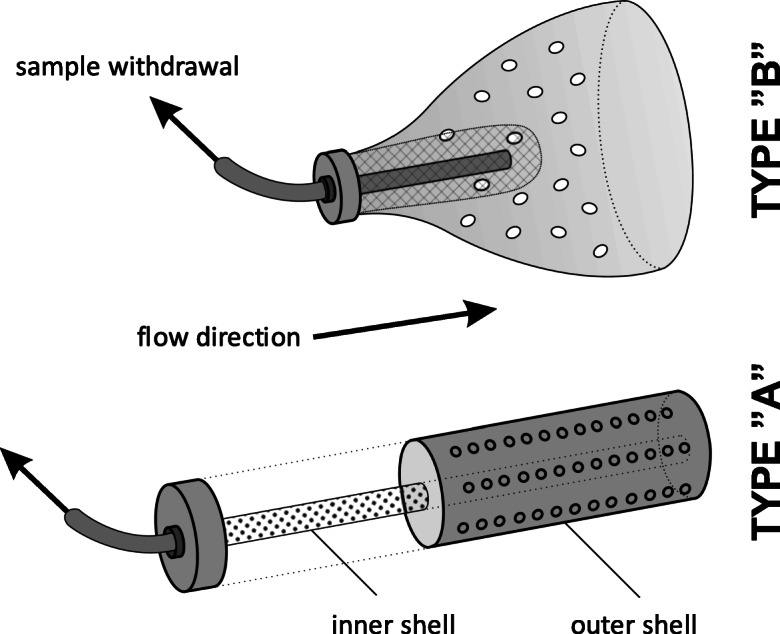
Structure of types “A” and “B” intake units (the former is shown in a dismantled state for clarity)

The sample intake unit serves a double purpose: (i) keeping entities larger than the internal diameter of the sample transmission tube (i.e., macroparticles, vegetation residues, etc.) off from entering the system, while (ii) enabling water and microparticles to pass through as freely as possible. As there is a strong trade-off between reaching these goals, especially during high-flow conditions, two different types of sample intake designs were evaluated during the field tests (Fig. 5). Type “A” intake consists of two concentric perforated plastic tubes: the outer shell, sealed permanently on the downstream side, features larger pores (3 mm), while the inner one, connected to the sample transmission tube by a push-in fitting on the upstream side, features smaller pores (1 mm). The two parts, when assembled, form a closed “shell-within-a-shell” structure. Type “B” intake consists of a perforated plastic cone, which is fully open on the downstream side and closed on the upstream side (where the sample transmission tube is connected by a push-in fitting). The 4-mm holes enable the flowing water to drag through the wall of the cone, and exit through the open side, allowing for turbulent mixing to take place within. This structure also serves as an effective spacer between the bottom of the sampled watercourse and the sampling tube inlet, while keeping the potential sedimentation low within the unit (especially compared with the type “A” intake unit). Inside the cone, another short tube section is adjoined to the push-in fitting and is surrounded by a 1-mm plastic mesh bag for protection against macroparticles.

Water from the sample intake is channeled through a weatherproof 6/4 mm (outer/inner diameter) SH A 98 polyurethane tube to the pumping unit, from where it travels through the same type of tube to the sample container. The water volume stored within the system (generally to be kept as low as possible, as it introduces some distortion to the final sample) depends on the overall length of these tubes.

### Software

**Fig. 6:**
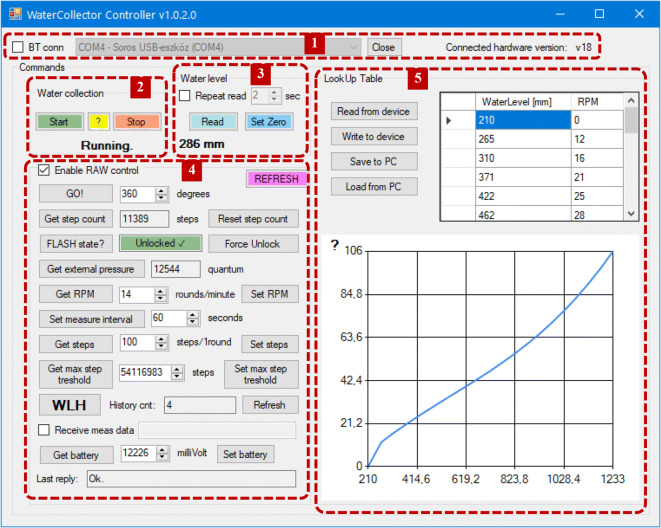
The main window of the Windows GUI

A computer program developed for Windows OS provides user-friendly control of the sampler. The graphical user interface (Fig. 6) features the following blocks:

1. Connection panel: for establishing or ceasing connection between the device and the PC
2. Main command panel: for starting and stopping the data/sample collection program on the device, as well as retrieving the current status of the program
3. Water level monitoring command panel: for retrieving the current measured water level and calibrating the WLT
4. Control command panel: for setting and reviewing various system constants and parameters, retrieving recorded data and manual control of the pump
5. Sampling curve panel: for defining, loading, and saving the lookup table (LUT) that specifies the sampling dynamics via corresponding pairs of water level and pump rotation values (expressed in mm and rounds per minute, respectively)

The number of data pairs in the LUT is limited up to 32 pairs, with a restriction that only integer values are allowed. The initial water level value, which triggers/ends the sampling session, is recommended to be set slightly above the base flow level of the sampled watercourse (this is always site specific and might need to be updated regularly to reflect seasonal variations). The water level in the last row of the table is respective to the highest flow value that the device will sample flow proportionally (higher flows will also be sampled but only with the fixed maximal pumping rate). The corresponding rotation speed depends on the achievable maximal sampling rate of the PU at the actual location (as described earlier) and the user-defined fixed ratio between the flow in the watercourse and the sampling rate (ensuring the flow proportionality of the sampling). The shape of the sampling control curve described by the LUT shall resemble that of the level vs. flow relationship valid at the actual site. Thus, in order to establish a proper LUT, the level-flow function must be known (or surveyed a priori) and the user shall pick an f-value that will accommodate the sampling rates into a range whose upper end matches with the full capacity of the pump at the chosen site. It must be noted that as the relationship between the water level and the sampling rate is defined by mutually corresponding points, the system cannot handle the hysteresis effect (when the rising and falling limbs of the hydrograph exhibit significantly different flow-level characteristics). However, if hysteresis is not pronounced (which is often the case in the targeted field of application), this limitation is not a disturbing factor.

A simple yet important feature of the program is that the maximum volume of the composite sample can be precisely set using the “max step threshold” value, which can be adjusted according to the available/desired sample storage capacity. This way, the device can be prevented from running longer than the volume of the sample container lasts.

**Fig. 7:**
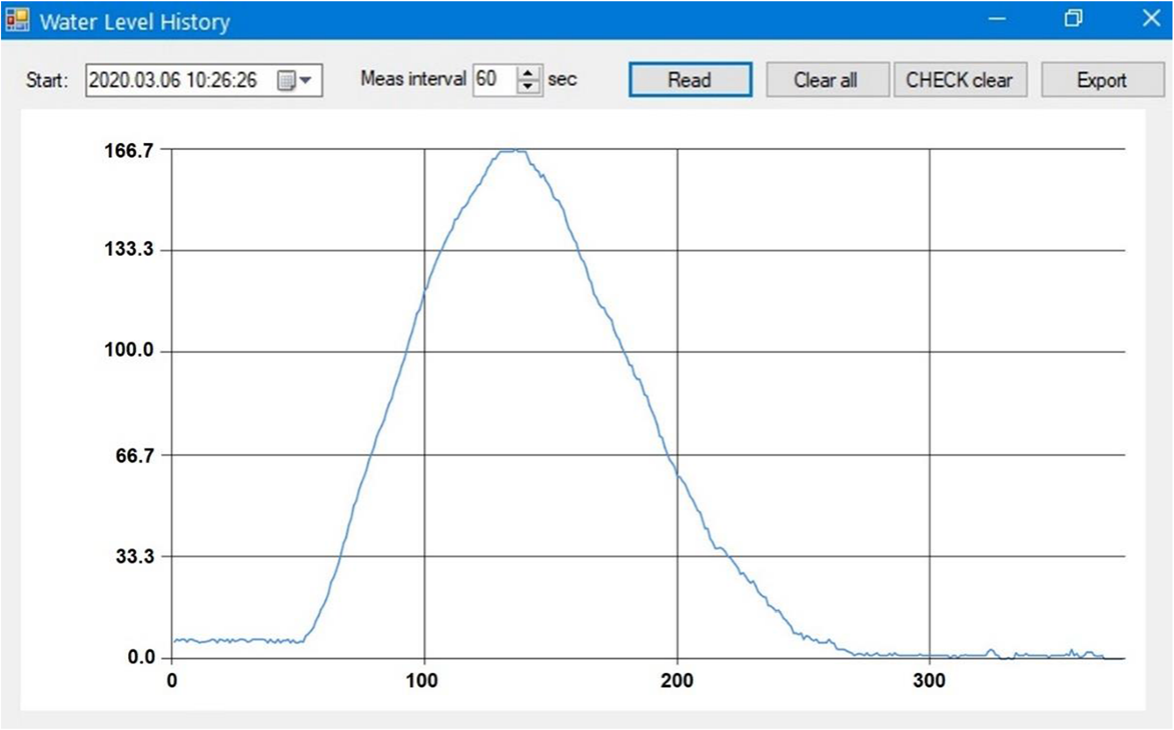
An example water level history plot window

When the sampling program on the device is in “running” mode, the firmware on the device keeps track of two parameters, which can be retrieved by the user program. The “step count” parameter is a single value, giving information on how much the pump has run in total since the program was last launched. Along with that, the device also registers a time series of the measured water level in intervals specified by the user on the control command panel (available down to 1 s). This time series, called “water level history” (WLH), can be plotted and exported as comma-separated values for subsequent data processing (Fig. 7). The count of registered data points is retrieved upon startup and presented on the control command panel for providing quick information. The maximum size of the WLH register is 46,080 data points (if data collection frequency is set to 1 min, this equals exactly to 32 days). Both the “step count” value and the WLH can be erased by the user at any time, when the program is in “stopped” mode.

For debugging purposes, a log file is always created on the connected PC upon the start of the user program. This log file registers all actions (commands and responses), as well as all data exchange (WLH reads and exports, LUT loads, and saves) of the session. Serving the convenience of the field personnel, a battery voltage check is also provided by the program (and automatically executed upon connection) in order to inform about the current charge level of the batteries which helps deciding whether an exchange is timely or not.

### Field testing

Sampler prototypes were deployed at seven urban sites in Hungary for several months. The selected catchments extended in size from a few blocks of streets to almost entire towns (30,000–100,000 inhabitants), representing a wide range of environments, including urban streams and stormwater drainage pipes as well (Table 2). The aim was to verify the overall applicability and to assess the capabilities of the developed device under different field situations.

**Table 2:** Characteristics of the seven urban sites where prototypes were tested

Site ID	Settlement	Channel type	Flow range	Lift height	Test period
GD	Gödöllő	Stream (natural bed)	50–1400 L/s	2.1 m	9 months
OA	Budapest	Stream (concrete bed)	5–330 L/s	1.0 m	8 months
ST	Salgótarján	Stream (paved bed)	30–1600 L/s	2.4 m	7 months
IF	Budapest	Stormwater pipe	5–1050 L/s	2.7 m	6.5 months
TB	Törökbálint	Stormwater pipe	0–20 L/s	1.4 m	5 months
PM	Pomáz	Stormwater pipe	0–300 L/s	1.2 m	4 months
FV	Székesfehérvár	Stream (paved bed)	400–1600 L/s	3.6 m	3 months

The site-specific level vs. flow relationship functions were derived previously using Nivus PCM Pro portable ultrasonic flow meter devices. Data from multiple consecutive runoff events were recorded at each site to construct the rating curves (such as the one shown by Fig. 6), which were used to develop the corresponding LUTs for the sampler program. Pump performance range was measured on the site at each location and was regularly checked and adjusted during the test period, when it was necessary. Water level logging was set to register data at 60-s intervals; consequently, when the pumping was active, the sampling rate was also updated automatically in every minute during runoff events.

**Fig. 8:**
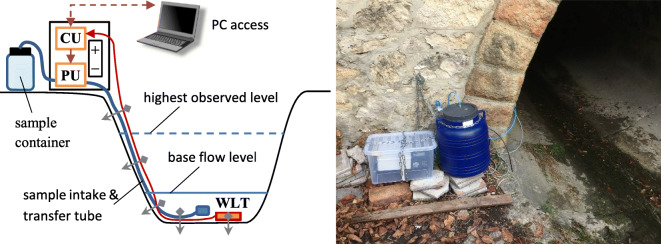
Typical system arrangement scheme at stream sites (communication cables are denoted by red arrows; fixing ties are symbolized by gray arrows), illustrated by a photograph from site OA

Figure 8 shows a typical example of how the system units were arranged at stream sites. The CU and PU were placed into a container box along with the external batteries, for extra protection from dirt and easier handling. The sample container was placed next to this box, at a safe height above the highest observed water level. The sample transmission tube and the communication cable from the WLT were routed along the bottom and the side of the channel, fixed regularly to the ground/bed with appropriate ties. Contrary to the WLT, which was mounted in a fixed way to remain in the same position all the time, some freedom of movement was allowed for the sample intake unit in order to be flexible during turbulent flows. In the case of stormwater pipes, the arrangement was similar but lacks the container box for the CU, PU, and external batteries, as these items had to be placed and secured individually, along with the sample container, to the wire steps of the access manholes (due to the very limited space available).

The field tests addressed two main questions: (1) how efficient and reliable the sample collection will be in general; and (2) how representative the collected composite samples will be for pollutants of interest. For the latter purpose, the total concentrations of 8 heavy metals (As, Cd, Cr, Cu, Ni, Pb, Sb, and Zn), total petroleum hydrocarbons (TPH), and 18 polycyclic aromatic hydrocarbon (PAH) compounds were analyzed in the samples, along with total phosphorus (TP). Heavy metals, TPH, and PAHs are regarded as typical pollutants of urban runoff (Aryal et al. 2010; Buzás et al. 2008; Göbel et al. 2007; Müller et al. 2020), while TP is of general interest. The studied 18 PAHs were the following (referred to as PAHs or PAH-18 from now on in this paper): naphthalene, 2-methyl-naphthalene, 1-methyl-naphthalene, acenaphthylene, acenaphthene, fluorine, phenanthrene, anthracene, fluoranthene, pyrene, benz(a)anthracene, chrysene, benzo(b)fluoranthene + benzo(k)fluoranthene, benzo(e)pyrene, benzo(a)pyrene, indeno(1,2,3-cd)pyrene, dibenzo(a,h)anthracene, and benzo(g,h,i)perylene.

In order to evaluate the representativity of the composite samples, series of manual grab samples were also taken during the course of five distinct runoff events (in parallel with the composite samples collected by the device) and, in addition, several times at base flow conditions as well. The intention was to compare the pollutant content of the runoff time series and the background grab samples with those of the composites. The role of the time series was to take snapshots of the contaminant composition from different stages of the runoff process and relate those to that of the composite, while the background samples were used to check against both types of runoff samples regarding the changes in the composition characteristics.

### Sample handling and analytical procedures

Samples were moved from the field to the laboratory as soon as possible after the rainfall events (which were monitored by following radar images published online by the Hungarian Meteorological Service in 10-min intervals). Cross-contamination between the consecutive samples was avoided by filling up the system from the base flow after recollection of the previous sample (the small distortion in concentration introduced by this step was accounted for later upon data processing, but proved to be generally negligible, due to the relatively large sample volumes). The collected samples were carefully partitioned according to the needs of the further analyses: for heavy metals and phosphorus determination, 100 mL of homogenized subsamples were transferred into PE flasks pre-acidified with 0.5 mL cc. HNO_3_; for TPH and PAHs, 1.5 L of homogenized subsamples were transferred into clean glass flasks. Subsamples were stored in a refrigerator at 4 °C until their chemical analysis, which took place in an accredited external laboratory.

In the laboratory, subsamples for total heavy metals and total phosphorus were digested using a Milestone UltraWave microwave digester, and quantitative analysis was executed by an Agilent 7500c ICP-MS device, according to the EPA 6020B:2014 method, using internal standards. TPH and PAHs were determined according to the following procedure. The volatile fraction of TPH was separated from the subsamples by the “purge and trap” method, using N_2_ as purging gas and activated carbon as adsorbent. The extractable fraction of TPH, along with the PAHs, were separated from the subsamples by liquid-liquid extraction, followed by elution in a chromatography column containing activated silica gel, using organic solvents (dichloromethane, hexane). Quantitative determination of PAHs was executed by GC-MS (using HP 6890 N gas chromatograph and HP 7683 automatic liquid sampler in conjunction with HP 5973 N MSD mass spectrometer), while GC-FID was applied for TPH (using HP-PONA gas chromatograph in conjunction with a flame ionization detector), in both cases with the help of internal standards.

## Results and discussion

**Fig. 9:**
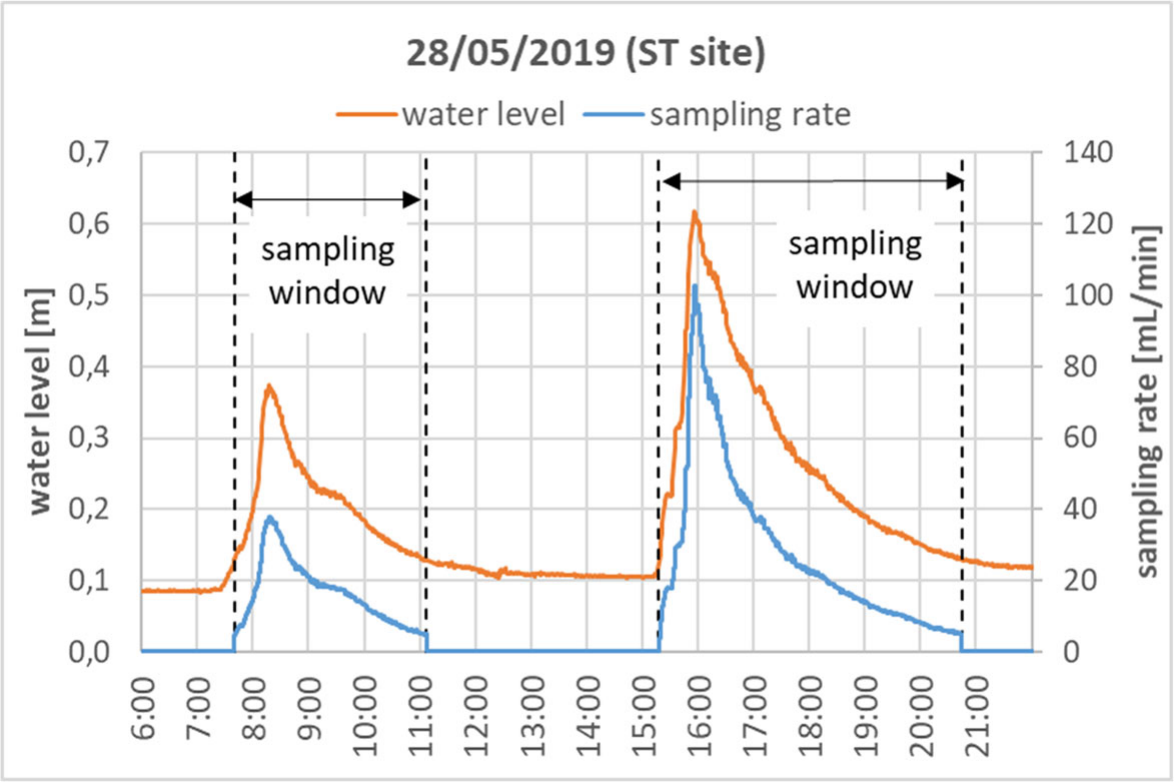
Example demonstrating the sampling characteristics for two consecutive runoff events at site ST

During the test period, a total of 97 automatic composite runoff samples were successfully collected from the seven sites, along with 28 manual grab samples—as time series—from five runoff events at two sites. An example hydrograph with the corresponding sampling rate history is presented by Fig. 9 for two consecutive events at the ST site. In this specific case, the sample collection time was 3.5 h for the first and 5.5 h for the second wave, covering the majority of both the rising and falling limbs of the hydrograph. The composite sample from the first wave had been transferred safely from the container before the rain that triggered the second wave started, and therefore the samples from two events could be separated from each other.

**Fig. 10:**
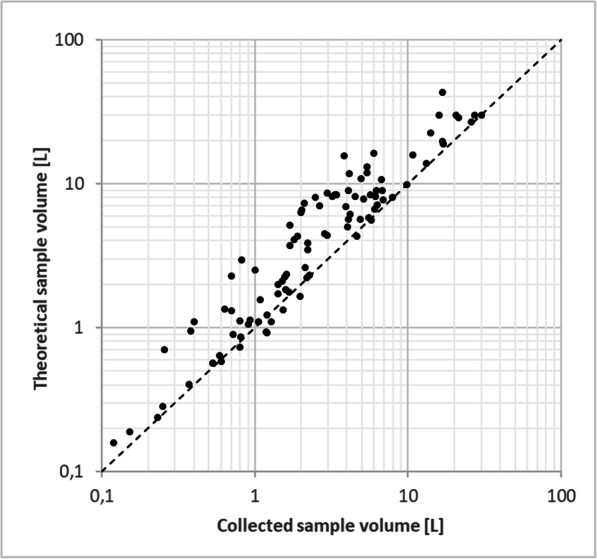
Theoretical vs. collected sample volumes. Dashed line represents the ideal 1:1 line

**Fig. 11:**
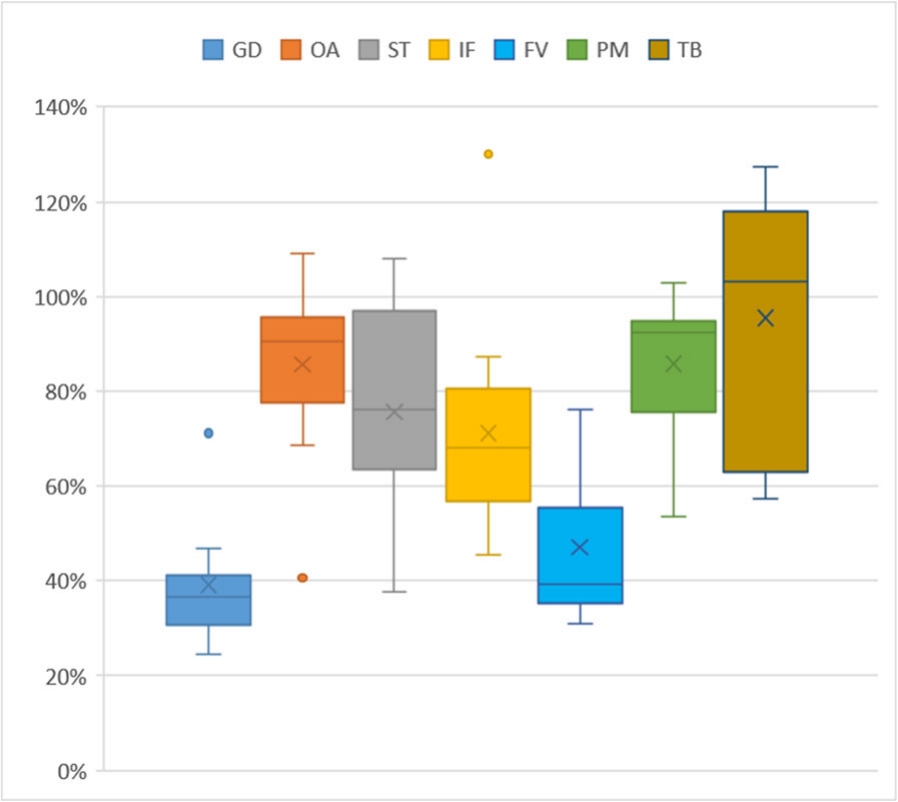
Box-and-whisker plots of sample collection efficiencies at the different test sites

The variety of the sites and events resulted in a wide range of composite sample volumes: from a few dL to 30 L (the latter being the upper limit defined by the sample container size). In order to evaluate the sampling efficiency, collected volumes were plotted against the theoretical ones (calculated from the registered “step count” values and the site-specific pump performances) on Fig. 10. Apparently, a large number of events were sampled with acceptable efficiency throughout the entire sample size range, while several others happened to exhibit considerable losses. The overall mean efficiency in the whole population was 71% with ± 26% standard deviation. Figure 11 and Table 3 provide further insight into the variability of sample collection efficiency, revealing that not only did this value vary among the individual events, but substantial differences can be observed between the test sites as well. In particular, two sites produced poor sample collection efficiencies, while the performance at the rest of the sites was in an acceptable range. However, a few events with low sample collection efficiency occurred at almost all sites.

**Table 3:** Variation of mean sampling efficiency and standard deviation among the different test sites

Site ID	GD	OA	ST	IF	TB	PM	FV
Mean	39%	86%	76%	71%	95%	86%	47%
STD	13%	15%	21%	23%	28%	15%	16%

Investigation of the reasons leading to undersampling confirmed that high loads of coarse-suspended sediment (sand and other heavy particles) present in the runoff tend to settle out within the sample intake unit (especially type “A”) due to reduced flow velocities, gradually impeding successful sample transmission, generally during the falling limb of the hydrograph. Intake units were therefore regularly checked and freed from debris, while sample transmission tubes were cleaned as well (using a portable vacuum pump) upon visiting the sites, whenever the circumstances allowed. These maintenance actions generally improved the sample collection efficiency. Replacing the intake units with type “B” designs also helped to enhance the situation. The case of site GD, however, was unique: due to the geography and the soil type of the watershed, the sampled creek produces extraordinarily high sand loads during runoff events, and despite the rigorous cleaning routine, as well as the application of type “B” intake unit, the issue persisted throughout the entire test period. Occasional clogging of the pump fittings due to fibrous plant residues also explained sample volume loss for a few events.

**Fig. 12:**
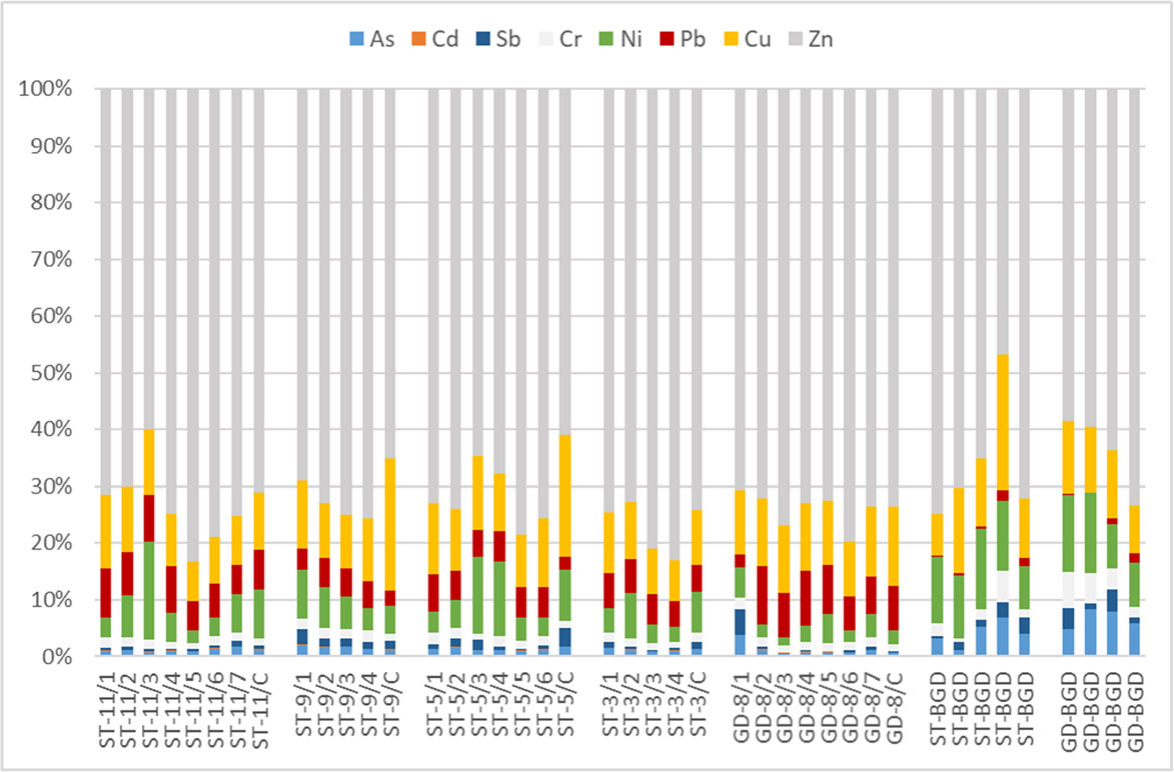
Relative amounts of heavy metals in the manual grab sample time series (ending in consecutive numbers) and the respective composite samples (last column in each block) from five distinct runoff events, in comparison with the background (last 9 columns), at two test sites (ST and GD)

The representativity of the composite samples collected by the device was evaluated by comparing the relative amounts (concentration ratios) of various pollutants in the following three sample groups: (1) grab sample time series taken during the course of five distinct runoff events at two sites, (2) the respective composite samples from the same events, and (3) manual grab samples taken at different times during base flow conditions. Figure 12 confirms that the relative amounts of heavy metals in the composite samples resemble quite well the general pattern exhibited by the distinct time series grab samples, while both groups are different from the base flow conditions.

**Fig. 13:**
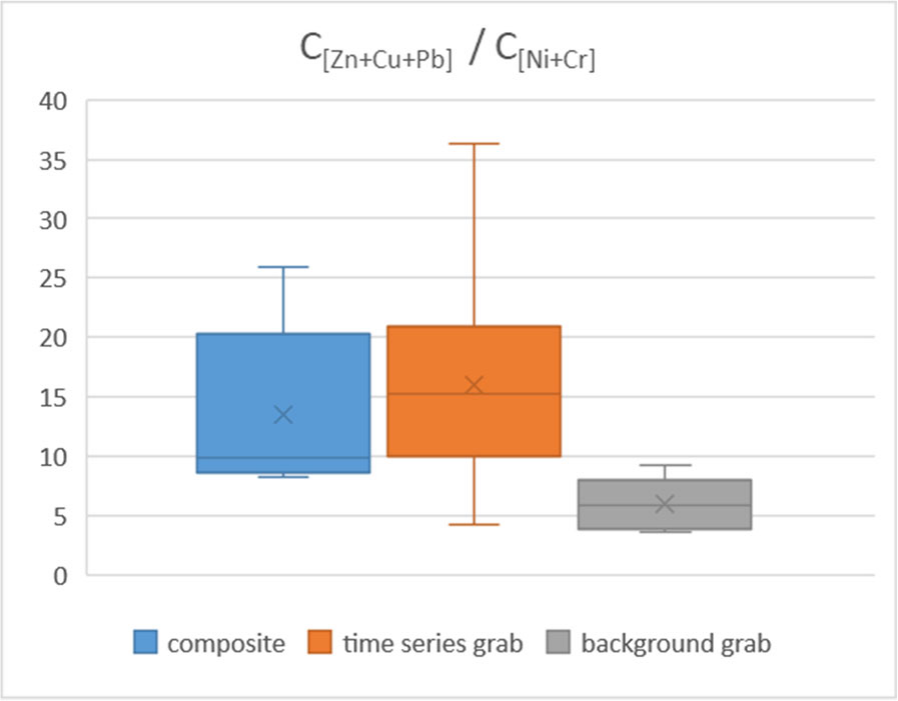
Box-and-whisker plots of the relative amounts of selected heavy metal groups in the different sample type groups of the sample set presented by Fig. 12

In general terms, the concentrations of all investigated pollutants increased from the background levels during runoff events, but the extent of the changes was more pronounced for some constituents than for the others. Such “enrichment” effect can be useful to demonstrate the representativity of the samples in a very simple form. In order to illustrate this, we took the sample set presented by Fig. 12, and for each of the samples, summed the measured concentrations of Pb, Cu, and Zn (featuring strong concentration changes during runoff, denoted by C_[Zn + Cu + Pb]_), and divided this with the sum of Ni and Cr (being less dynamic, denoted by C_[Ni + Cr]_). The heavy metals of the former cluster are well-known urban stormwater pollutants, typically originating from vehicle brake and tire wear emissions, as well as roofing material weathering, while the latter cluster features heavy metals which are abundant in urban runoff but their sources are less specific. Figure 13 presents the distribution of the resulting values in the three sample type groups (composite, time series grab, and background grab) as box-and-whisker plots. Apparently, the composite samples resemble well the central bulk of the time series grab samples (which were scattered along the entire time span of the runoff events, resulting in higher variability that is reflected by the wider range of the values), while both are significantly different from the typical base flow conditions.

**Fig. 14:**
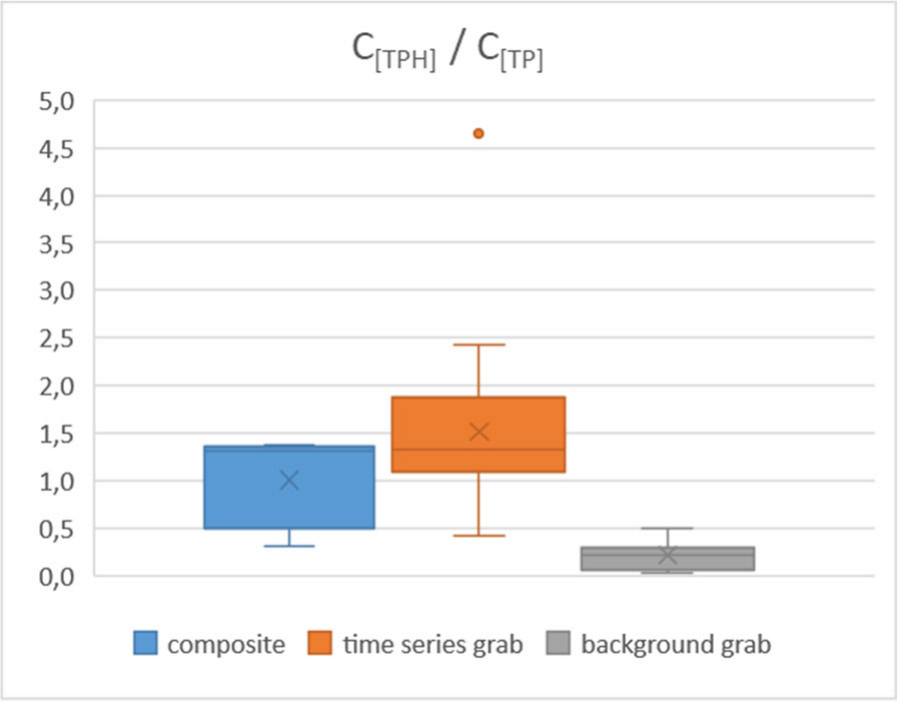
Box-and-whisker plots of the relative amounts of total petroleum hydrocarbons vs. total phosphorus in the different sample type groups of the sample set presented by Fig. 12

**Fig. 15:**
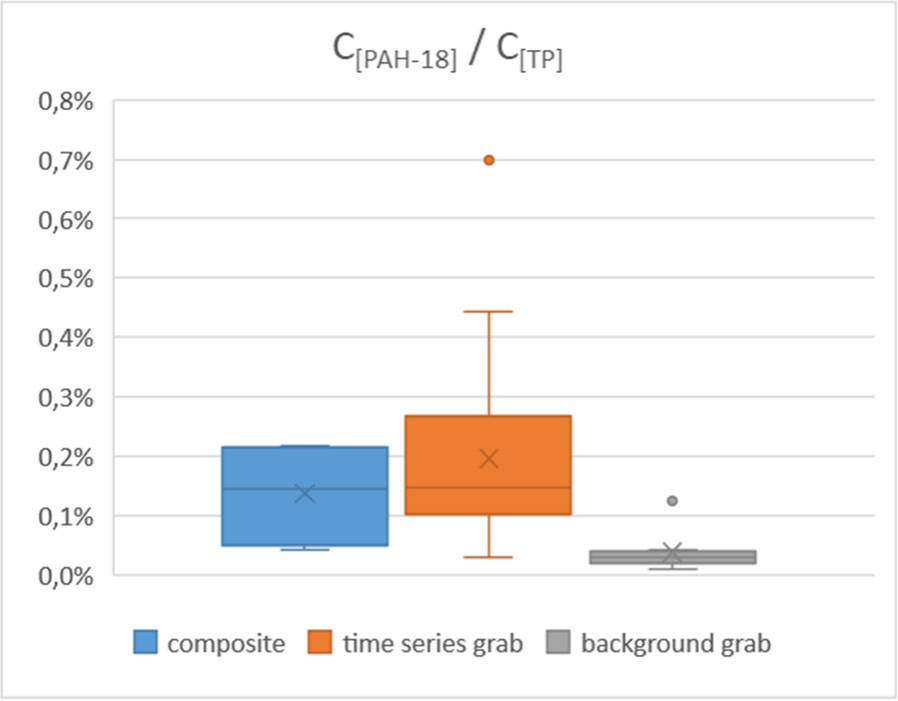
Box-and-whisker plots of the relative amounts of the studied PAH compounds vs. total phosphorus in the different sample type groups of the sample set presented by Fig. 12

Following a similar line of thought, the relative amounts of TPH and PAH-18 to TP were also investigated, in order to provide further evidence for the representativity of the composite samples. Again, the former pollutants are typical urban organic contaminants, while the latter is a rather general one but also present in urban runoff. Comparison of the aforementioned concentration ratios (denoted as C_[TPH]_/C_[TP]_ and C_[PAH-18]_/C_[TP]_) in the three sample groups (Figs. 14 and 15) reveals a similar picture to what was presented earlier with the selected heavy metals, although with some differences between the distributions of the values for the composite and time series grab samples. This can be explained by the circumstance that a couple of data points (including the outliers) in the latter group represent a more pronounced “first flush effect” (a term widely used to describe outstanding pollutant concentrations at the initial stage of the runoff events—see, e.g., Bertrand-Krajewski et al. 1998) for both TPH and PAH-18. The respective distributions are thus slightly skewed towards the higher values but are nevertheless indicating that the characteristics of the runoff samples (composite and the time series grab) are identical to each other, while at the same time both are certainly distinct from the background flow grab samples.

Based on the findings above, we infer that the sampling method employed by the autonomous device presented in this paper proved to be sufficiently reliable and the collected composite samples are representative concerning micropollutants which are present as, or bound to, fine and colloidal particles in urban runoff.

## Conclusions

The widely customizable, yet relatively inexpensive autonomous flow-proportional water sampler assembly described in this paper proved to function reliably for prolonged times in a wide range of different environments. It has been shown that the concept is suitable for conducting cost-effective urban runoff characterization surveys targeting inter-event variability. The developed system is flexible enough to be adapted to various deployment requirements and is able to deliver representative composite samples for pollutants specific to the urban environment. Sample transmission from the watercourse to the sample container can pose challenges, depending on site properties such as the height and length that must be overcome, as well as the composition of the runoff. Regular system checks and maintenance activities are therefore necessary, especially at sites where the coarse fraction of suspended solid loads is high during runoff events.

Further development of the current prototype is encouraged. Communication abilities could be extended with the addition of a low-power, wide-area network module (e.g., LoRa or NB-IOT), a wireless technology capable of transferring small data packages intermittently that could be used to acquire the latest water level and step count data remotely from the device. Improvements are also possible regarding the sample transmission system, e.g., by upgrading the pumping units with stronger pumps to enhance sampling velocities (which would also necessitate larger sample containers) or by designing more advanced sample intake units.

## Data Availability

Not applicable.
